# Implementing a free lending of sports and leisure equipment service: a cross-sectional survey exploring user characteristics, utilization patterns, and significance among children and youth

**DOI:** 10.1186/s12889-024-19339-3

**Published:** 2024-07-09

**Authors:** Johan Högman, Stefan Wagnsson, Sebastian Bellander

**Affiliations:** Department for Educational Work, Sport Science, Karlstad University, Karlstad, Sweden

**Keywords:** Sports equipment, Youth physical activity, Socioeconomic disparities, Brand identification, Nonprofit initiatives, Equipment lending

## Abstract

**Background:**

The escalating costs of sports equipment, coupled with socioeconomic disparities, hinder children’s participation in physical activities. The Leisure Equipment Library (LEL), a unique service in Sweden, addresses this challenge by providing free equipment lending. This study investigated the significance of providing free lending of sports and recreational equipment for children’s and youth’s opportunities to engage in physical activities during leisure time.

**Method:**

Utilizing a cross-sectional survey during the summer-2022 period, the study is based on data from 427 LEL users aged 7 to 25 years. User characteristics (demography, socioeconomic status, physical activity profiles), equipment usage patterns and perceived significance are also analyzed. The findings are discussed using the concept of consumer-brand identification.

**Results:**

The results showed that LEL reaches a broad and diverse group of children and young people in terms of gender, age, socioeconomic status, and physical activity profile. The borrowed equipment, primarily used for play and recreation, serves as an essential resource for children and youths and, in particular, for those with low socioeconomic status. LEL is considered highly important by all user groups, with a majority emphasizing its significance in facilitating leisure activities.

**Conclusions:**

The study suggests LEL’s success lies in its accessibility and that users identify with the brand of LEL. Users perceive LEL as a service catering to various recreational needs rather than sports, fostering inclusivity. The localization of stores in various types of areas, combined with high-quality products, enable children and young people from different socioeconomic areas to use the service. LEL’s success hinges on maintaining a positive brand image and promoting a recreation-oriented identity. Opportunities exist to strengthen emotional bonds with users, enhance branding strategies, and position LEL as a valuable resource for inclusive recreational activities. In conclusion, this study highlights the potential of free lending services, such as LEL, to bridge socioeconomic gaps in the promotion of physical activity among children and young people.

**Supplementary Information:**

The online version contains supplementary material available at 10.1186/s12889-024-19339-3.

## Introduction

Efforts to promote physical activity (PA) among children and youth are recognized by the World Health Organization [1] as one of the key areas within health promotion work in the future. System-based models emphasize the significance of addressing how factors within different societal systems interact with and impact opportunities for individuals and groups to engage in PA [2, 3]. Active leisure time has been highlighted as one of the key areas, with particular emphasis on striving for equitable access to sports and recreational activities. For example, outdoor play can reduce sedentary behavior later in life [4].

Access to equipment is a fundamental requirement for children and youth to participate in sports and recreational activities. Equipment is, after fees, the largest cost component for individuals involved in sports. In 2021, the average Swede spent $250 on sports equipment (including clothing) [5]. The costs of various sports and activities vary greatly, but the need for equipment is universal, and it is becoming increasingly expensive. The average American family pays $883 annually for one child’s primary sport. Of this, $154 is for Equipment. Equipment costs are the next most significant reason for increasing costs for youth sports in the USA [6].

Numerous studies have demonstrated the impact of socioeconomic factors on children’s PA, even in high-income countries [7, 8]. This influence is particularly pronounced among girls, making them a prioritized group for supportive interventions [8]. Socioeconomic structures affect children’s and youth’s PA in complex ways, with research suggesting that economic factors play a significant role. For instance, a study conducted in Belgium revealed a greater likelihood of exclusion from organized sports among children from disadvantaged households whose parents had low educational attainment [9]. Similarly, Andersson and Bakken [10] found that economic, rather than social, capital predicts participation in organized sports in Norway. Unstructured PA, in turn, generally declines with age, but research indicates that children (aged 11–13) with higher socioeconomic status experience a less pronounced decline [11]. To find promotive solutions, studies have examined whether various forms of fee subsidies can contribute to reducing inequalities in PA engagement [12]. Reece et al. [13] showed that a voucher programme, including reduced participation fees, may increase participation among children and youth given that such programmes are designed in a way that reaches and is utilized by children and youths with low socioeconomic status. In the same vein, a study by Reilly et al. [14] of a voucher programme (Active Kids scheme) aimed at promoting participation in organized sports in Australia showed that the programme did not yield the desired effect among the target groups that need it the most: socioeconomically disadvantaged individuals and children and youth with the lowest PA levels. Instead, the vouchers were utilized by more affluent families that were already physically active. Different tax reduction programs have shown some promise [15], although their effectiveness is contingent on the structure of the system [16]. However, a recurring issue, even in this regard, is that children and families with lower socioeconomic status often fail to take advantage of such subsidies [12]. Another highlighted concern is the stigma associated with complex application processes for accessing subsidies [12]. Consequently, research has yet to demonstrate satisfactory policy interventions to reduce financial barriers to PA for children and young people. Thus, there is a pressing need to continue examining efforts aimed at enabling more children and youth to participate in sports and recreational activities and reducing disparities in PA levels. Surprisingly, few studies have focused specifically on equipment costs as a barrier to engaging in physical activities. Despite fee subsidies, parents often highlight the significant remaining expenses associated with children’s equipment. In many cases, parents are left to find individual solutions, such as swapping equipment among themselves or inheriting from older relatives [12]. Free lending of equipment, for instance, as provided by the Leisure Equipment Library (LEL) examined in this study, can potentially function as a solution to this problem. However, to determine how such an intervention would be useful in practice, it is important to examine who utilizes this service and how it may contribute to increased PA. Therefore, the purpose of this study was to investigate the importance of providing free lending of sports and recreational equipment for children’s and youth’s opportunities to engage in physical activities during leisure time.

For more than 10 years, Sweden has had a service called the Leisure Equipment Library (LEL), which offers free lending of sports and leisure equipment. An LEL can be likened to an ordinary library but for sports and recreational equipment. Most LEL facilities have thousands of items in stock, ranging from balls, football boots, skis, and ice skates to outdoor gear, bicycles, and life jackets. According to their own database, the LEL has lent out 3.8 million items since the start of 2013, with a 50% increase from 2023 compared to 2022. Individuals can visit an LEL to browse, try out, and borrow equipment free of charge for a period of 14 days. The equipment is preowned, and LELs receive their items from recycling centers or people directly donating their used equipment to their nearest LEL. In recent years, it has also been common for elite-level athletes and teams to donate high-quality equipment. At LELs, staff repair and ensure that the equipment is in a lendable condition. According to the organization, this circular thinking is an important part of their operation, aiming to both protect the environment and keep costs down. The central organization is a nonprofit organization (NPO), while nine out of the ten local LEL units are financed and operated by municipalities. The relation between the local units and the central organization can be compared to that of a franchise; however, the difference is that the use of the brand is not associated with any costs. In the summer of 2023, 125 units have been established all over Sweden. In the coming years, approximately ten new units are expected to open annually. Approximately one-third of all LELs are located in areas facing socioeconomic challenges, with the majority concentrated in smaller or medium-sized urban areas. Almost half of the LELs are located in socioeconomically mixed areas, while the remaining half, approximately every fifth LEL, are located in areas with favorable socioeconomic conditions [17].

## Consumer-brand identification

The foundation for the use of LEL is likely rooted primarily in economic aspects, as it involves the challenge of high equipment costs, which we described earlier. However, we argue that this does not provide the entire explanation of usage. Consequently, to understand why certain individuals and groups choose to use (or not use) LELs, we employ the concept of consumer-brand identification [18]. According to consumer brand identification theory, people use services with which they can to some extent identify. The basic premise behind the idea that people identify with brands is rooted in social identity. This theory posits that individuals group within society to achieve meaningful self-definition through intergroup homogenization and intragroup differentiation [19, 20]. Connecting to various brands is a part of this group-formation process. Bhattacharya and Sen [21, p. 77]. have argued that people’s identification with an organization is motivated by the ‘satisfaction of one or more self-definitional (i.e., ‘Who am I?’) needs’. We argue that the service offered by LEL is associated with a specific brand, in a broad sense. The LEL, through their logos, their marketing, and, of course, their service, symbolize something that users perceive in particular ways. Using an LEL service involves, in a sense, identifying oneself with what its brand represents [18, 21]. In the marketing literature, service quality is emphasized as a crucial component of customer satisfaction, which in turn leads to increased loyalty [22, 23]. This is also a crucial aspect of LEL, as it provides a free service based on donated and second-hand products. Furthermore, many individuals who are distant from the labor market and who participate in public support programs engage in work training within the organization, which also influences the quality of service. Another significant aspect of LEL is that its operations are not driven by for-profit motives; rather, it belongs to a public and nonprofit sphere. Aaker et al. have shown that NPOs are perceived as warmer but also less competent than for-profit brands [24]. This would produce feelings of pity among the general population. However, it is also possible that people from lower socioeconomic groups, based on their limited economic resources, may perceive NPOs as important and trustworthy, which then produces positive feelings toward the brand.

In this case, we argue that using an LEL is also strongly linked to a person’s relationship with PA. The relationship between a brand and an individual is built not only on the character of the brand but also on the individual’s characteristics. Using the LEL is associated with an expectation of engaging in physical activities. Research on PA behavior has shown that it is necessary to some extent to identify oneself as an active person to be inclined to engage in PA behaviors [25, 26]. The development of such self-perception is closely linked to one’s perceived competence in engaging in physical activities [27, 28]. However, identifying oneself as an athlete differs somewhat from participating in more playful and recreational physical activities [26]. Involvement in sports entails greater demands on skills and mental attributes such as persistence and a competitive spirit. While many children and young people enjoy various physical activities, they may not necessarily encounter these specific demands in their pursuits [29].

## Method

### Study design and procedure

This study was designed as a cross-sectional survey targeting the population of users of the LEL aged 7 to 25 years. The age group adheres to an established administrative classification within the leisure sector in Sweden, where individuals aged 7 to 25 years are categorized as ‘children and young people.’ This classification is also the target group for government subsidies. It was deemed most appropriate to reach users directly when they visited an LEL. The CEO of the central organization of the LEL was contacted to distribute the study invitation to all 123 LELs. Two informational meetings were held where the researchers provided detailed information to the staff regarding the study objectives and data collection procedures. A research assistant was available on a daily basis to address any queries raised by the staff responsible for survey distribution. While participation in the study was voluntary, every LEL was encouraged to assist with the data collection. Surveys, along with comprehensive instructions, were sent electronically to all LELs. It was explicitly communicated that respondents had the option to complete the survey digitally or on paper and that the survey was available in both English and Swedish. Younger children who experienced difficulties reading the questions were encouraged by survey distributors to seek assistance from parents or staff when completing the survey.

### Sample

Out of the 123 LELs, 52 successfully collected at least one completed questionnaire during the specified data collection period, which spanned from June 1st to September 15th, 2022. The nonparticipation of certain LELs can be attributed to various reasons, such as temporary closure during the designated period, unavailability of willing users to participate in the survey, or a deliberate choice not to engage in the study. In total, 521 individuals responded to the survey. After thorough evaluation for completeness, 94 partially completed surveys were excluded, resulting in a final survey sample of 427 participants for the study.

### Questionnaire

A new questionnaire was constructed for this study (see Additional file 1). The questionnaire consisted of a total of 25 items. Demographic information (i.e., gender, and age) was assessed using single-item questions. As an indicator of socioeconomic status, users’ residential addresses were collected and coded according to a national database that categorizes residential areas into different socioeconomic categories [30]. The categorization was predicated on entities referred to as regional statistical areas, which delineate specific geographic regions. In Sweden, there exist 3,363 such designated areas. For each area, a socioeconomic index has been computed, drawing on three indicators (I): (1) the proportion of individuals with pre-secondary education, (2) the proportion of individuals with low economic standards, and (3) the proportion of individuals receiving financial assistance for at least ten months and/or being unemployed for longer than six months. The index was then calculated as (I1 + I2 + I3)/3. Subsequently, five area types were established based on the number of standard deviations from the mean of the index (areas with significant socioeconomic challenges: ≥ 2 standard deviations; Areas with socioeconomic challenges: ≥ 1 standard deviation and < 2 standard deviations; socioeconomically mixed areas: ≥ 0 standard deviations and < 1 standard deviation; areas with favorable socioeconomic conditions: ≥ − 1 standard deviation and < 0 standard deviations; areas with very favorable socioeconomic conditions: < − 1 standard deviation from the index average [30]. To examine whether the level of PA and physical competence were significant for whether the participants visited LEL, three PA profiles (PAP) (i.e., Low, Medium and High) were created through two-step cluster analysis (see further description below). The analysis was based on the following one-item questions related to participants’ (i) sports background (i.e., ‘Do you participate in any sports or athletic activities in a sports club?’; 0 = No, 1 = Yes); (ii) current leisure interests (i.e., ‘What else do you do in your leisure time?’—e.g., ‘I work out at the gym’; 0 = No, 1 = Yes); and (iii) current PA level (i.e., ‘How often do you engage in PA that makes you breathe heavily or sweat?’; 1 = Never, 6 = Every day). Finally, (iv) perceived physical competence was measured using two items from the ‘The Perceived Competence Scale for Children’ [28] (e.g., ‘Compared to others your age, are you better or worse at sports?’; 1 = Much worse, 4 = Much Better). Three additional one-item questions were included to gather information on borrowed equipment, including where with whom, and how respondents intended to use it. The significance attributed to the activity by the users was measured through two separate questions: ‘What would you have done if you could not borrow equipment from the LEL?’ and ‘How important is the LEL for you to be able to engage in activities during your leisure time?’. To ensure the readability and comprehensibility of the survey questions among the targeted population, two pilot surveys were conducted. Minor modifications in language were made after pilot testing.

### Analysis

To assess whether LEL users differ from the characteristics of the Swedish population, confidence intervals were calculated for each characteristic of LEL users. These values were subsequently compared to the known population values. In cases where the population values fell outside the confidence intervals of the sample, it was inferred, with a 95% confidence level, that the characteristics of LEL users significantly differ from those of the Swedish population. A two-step cluster analysis utilizing the log-likelihood measure was conducted in SPSS version 28.0 to delineate groups within the dataset based on their levels of PA and physical capability. A two-step cluster analysis was considered most appropriate since it allows the creation of clusters based on both continuous and categorical data [31]. To limit the number of clusters and consequently make the analysis more manageable and facilitate interpretation, a predetermined number of clusters was stipulated prior to conducting the analysis. The analysis revealed three clusters with a 1.56 ratio of sizes (Cluster 1: *n* = 113, 27%; Cluster 2: *n* = 32%; Cluster 3: *n* = 42%); a fair/good cluster quality (Avg Silhouette Score = 0.5); and importance values above 0.08 for all the input variables. A series of chi-square tests were subsequently conducted to examine potential associations between the characteristics of LEL users and the utilization of borrowed equipment in various activities. Additional chi-square tests were also conducted to examine potential associations between LEL user characteristics and both the neglect of activities due to the inability to borrow equipment from LEL and the significance of LEL in enabling their involvement in physical leisure activities. The significance level for all analyses was set at 0.05.

## Results

### Characteristics of LEL users

As presented in Table 1, a majority of the users are boys. The observed differences are relatively modest and are comparable to the national data referred to as ‘Characteristics of the Swedish population 7–25 years’ in the table. The average age of LEL users is 15 years. The distribution between the age groups shows a relatively uniform pattern, although in comparison with national data, there is an overrepresentation of users between 13 and 18 years of age, together with a corresponding underrepresentation in the 19–25 age group. The largest proportion of users (45%) reside in areas with good or very good socioeconomic conditions, while a slightly smaller proportion (29%) live in areas facing socioeconomic challenges. However, according to Swedish national data, slightly more than half of individuals aged 7–25 in Sweden reside in areas with favorable or very favorable socioeconomic conditions, while a significantly smaller portion reside in areas with socioeconomic challenges [32]. Consequently, when comparing the LEL sample with national data, there is an overrepresentation of children and young people from areas facing socioeconomic challenges. Although a relatively large proportion (59%) of individuals are involved in organized sports (i.e., sports clubs) and/or gym activities (41%), the largest proportion of users, categorized by their PAP, belong to the low-PAP group. This was followed by the Medium and High PAP groups, indicating that LEL was effectively reaching the group of children and youth most in need of PA.

**Table 1:** Characteristics of LEL loaners in relation to the Swedish population (7–25 years)

Characteristics of LEL Loaners (7–25 Years)				Characteristics of the Swedish Population (7–25 years)	
	*n*	%	95% CI	*N*	%
Gender^a^					
*Boys*	234	56	51–61	1 184 256	52
*Girls*	183	44	39–49	1 095 584	48
*Total*	417	100		2 279 840	100
Age^a^					
*7–12 years*	150	35	31–39	750 609	33
*13–18 years*	169	40	35–45	709 877	31
*19–25 years*	108	25	21–29	819 443	36
*Total*	427	100		2 279 840	100
Socioeconomic Residential Areas^a^					
*Significant Socio-* *Economic Challenges*	40	9	8–10	153 005	7
*Socio-Economic* *Challenges*	85	20	18–22	205 674	9
*Mixed Areas*	134	31	27–35	486 022	21
*Favorable Socio-* *Economic Conditions*	143	34	30–38	1 178 216	52
*Very Favorable Socio-* *Economic Conditions*	25	6	4–8	256 923	11
*Total*	427	100		2 279 840	100
Sports and leisure habits^b^					
*Organized Sports*	252	59	54–64		
*Gym*	175	41	36–46		
*Scouts*	47	11	8–14		
*Dance*	43	10	7–13		
*Video Game Association*	30	7	5–9		
*Religious Association*	30	7	5–9		
*Cultural Activities*	26	6	4–8		
*Nothing Particular*	116	27	23–31		
Physical Activity Profiles					
*Low*	176	41	36–46		
*Medium*	134	32	28–36		
*High*	113	27	23–31		
*Total*	423	100			

Note: ^a^ Numbers from 2020; ^b^ Several options were possible

### Equipment usage

Nearly one-third of all loans at LELs involve equipment for various street sports, such as cycling, inline skating, and skateboarding. Almost an equally significant share (27%) of the participants consisted of equipment for various team ball sports (handball, football, basketball, floorball, volleyball, etc.). Approximately 14% of all loans pertain to outdoor activity equipment, encompassing items such as tents, sleeping bags, sleeping pads, fishing gear, and similar items. The various types of racket sports and equipment for games/summer activities each account for approximately 7% of the total lending during the summer period. The most common purpose for utilizing borrowed equipment is to play with friends or family, as reported by just over half of the participants. The next most common use is for play or exercise alone, followed by spontaneous sports activities with others. Notably, only a small percentage of participants (13%) reported that they intended to use the equipment to participate in organized sports. When the utilization of borrowed equipment in various activities was compared to the characteristics of the participants, the results showed no significant differences between boys and girls in terms of borrowing patterns. Although significant differences in the intended use of the equipment could be observed within the groups related to age, socioeconomic residential area, and PAP, a deeper analysis of the adjusted residuals did not reveal any meaningful patterns (see Table 2).

**Table 2:** Utilization of borrowed equipment

Utilization of Borrowed Equipment					
Characteristics of LEL Loaners (7–25 Years)	Play with Friends or Family	Play/ Exercise Alone	Play/Exercise with Unfamiliar Individuals	Spontaneous Sports with Others	Organized Sports
	*n* (%)	*n* (%)	*n* (%)	*n* (%)	*n* (%)
Gender					
*Boys*	119 (52)	55 [24]	16 [7]	42 [18]	34 [15]
*Girls*	105 (57)	42 [23]	9 [5]	34 [19]	17 [9]
*Total*	224 (54)	97 [23]	25 [6]	76 [18]	51 [12]
*Χ*^*2*^/*p-value*	1.41/0.235	0.04/0.838	0.73/0.394	0.01/0.917	2.79/0.095
*Phi*	0.058	− 0.010	− 0.042	0.005	− 0.082
Age					
*7–12 years*	81 (55)	40 [27]	7 [5]	18 [12]	14 [10]
*13–18 years*	102 (61)	40 [24]	12 [7]	31 [18]	30 [18]
*19–25 years*	46 (43)	19 [18]	6 [6]	28 [26]	10 [9]
*Total*	229 (54)	99 [23]	25 [6]	77 [18]	54 [13]
*Χ*^*2*^/*p-value*	8.74/0.013*	3.14/0.208	0.86/0.652	7.97/0.019*	6.57/0.037*
*Cramer’s V*	0.144	0.086	0.045	0.137	0.124
Socioeconomic Residential Areas					
*Significant Socio-* *Economic Challenges*	13 [32]	6 [15]	4 [10]	11 [28]	9 [22]
*Socio-Economic* *Challenges*	53 (63)	18 [21]	5 [6]	13 [16]	14 [17]
*Mixed Areas*	75 (57)	36 [27]	6 [4]	32 [24]	18 [14]
*Favorable Socio-* *Economic Conditions*	72 (50)	36 [25]	9 [6]	15 [10]	11 [8]
*Very Favorable Socio-* *Economic Conditions*	16 (64)	3 [12]	1 [4]	6 [24]	2 [8]
*Total*	229 (54)	99 [23]	25 [6]	77 [18]	54 [13]
*Χ*^*2*^/*p-value*	12.44/0.014*	4.93/0.294	1.85/0.763	12.28/0.015*	8.47/0.076
*Cramer’s V*	0.171	0.108	0.066	0.170	0.141
Physical Activity Profiles					
*Low*	92 (52)	42 [24]	5 [3]	27 [15]	9 [5]
*Medium*	65 (58)	27 [24]	12 [11]	31 [27]	27 [24]
*High*	71 (54)	28 [21]	8 [6]	19 [15]	17 [13]
*Total*	228 (54)	97 [23]	25 [6]	77 [18]	53 [13]
*Χ*^*2*^/*p-value*	0.77/0.682	0.32/0.853	7.45/0.024*	8.59/0.014*	22.03/<0.001**
*Cramer’s V*	0.043	0.027	0.133	0.143	0.229
Total	229 (54)	99 [23]	25 [6]	77 [18]	54 [13]

^1^ Multiple answers were possible; *p < .05, **p < .01

### Perceived significance of LEL for sports and physical leisure activities

When participants were asked to assess the importance of the LEL in facilitating their participation in sports and physical leisure activities, 62% reported that the LEL is important or very important to them. Only 7% reported that the LEL service was not important at all. Subsequent analysis, pooling the response options (Not important at all/Somewhat important = 0, Important/Very important = 1), revealed no apparent association with gender (Females 67%, Males 59%, χ^2^ = 2.42, *p* = .120, phi = − 0.076), or PAP groups (Low PAP: 66%; Medium PAP: 65%, High PAP: 56%, χ^2^ = 3.40, *p* = .183, Cramer’s V = 0.090). However, significant associations were observed with age (7–12 years: 63%; 13–18 years: 55%, 19–25 years: 73%; χ^2^ = 9.25, *p* = .010; Cramer’s V = 0.148) and socioeconomic residential areas (Significant Socioeconomic Challenges: 78%, Socioeconomic Challenges 69%, Mixed Areas 63%, Favorable Socioeconomic Conditions 53%, Very Favorable Socioeconomic Conditions 68%, χ^2^ = 11.36, *p* = .023, Cramer’s V = 0.163), indicating that it holds relatively greater importance for older youths (19–25 years) and children and youths residing in areas characterized by significant socioeconomic challenges, to have the ability to borrow sports and leisure equipment for free. Across all demographic groups, half of the respondents indicated that they would have neglected the activity if they could not borrow equipment from LEL. Hence, LEL appears to stimulate numerous activities that might otherwise have been foregone. Among those who reported that they otherwise would have neglected the activity, a larger proportion included females (Females 56%, Males 45%, χ^2^ = 5.32, *p* = .021, phi = − 0.113); children aged 7–12 years (7–12 years: 55%, 13–18 years: 42%, 19–25 years: 53%, χ^2^ = 8.43, *p* = .015, Cramer’s V = 0.141); and individuals belonging to the Low PAP group (Low PAP: 56%; Medium PAP: 41%, High PAP: 50%, χ^2^ = 6.42, *p* = .040; Cramer’s V = 0.124), while no significant association was found between socioeconomic residential areas and neglecting the activity (Significant Socioeconomic Challenges: 48%, Socioeconomic Challenges 42%, Mixed Areas 51%, Favorable Socioeconomic Conditions 54%, Very Favorable Socioeconomic Conditions 60%, χ^2^ = 3.86, *p* = .425, Cramer’s V = 0.095).

## Discussion

A recurring challenge in broad public health promotion initiatives is the uneven utilization of interventions. For instance, individuals with low socioeconomic status are less likely to take advantage of public tax deductions related to PA [12]. This study demonstrates that, in the case of LEL, intervention to a greater extent reaches children and youth living in areas facing socioeconomic challenges. Part of the explanation is likely that many LELs are located in areas with socioeconomic challenges, thus increasing the geographical accessibility for that group. However, according to the concept of consumer-brand identification, users also need to identify with the product/service in question to feel compelled to use it [18]. In other words, there seems to be, at present, an acceptance among both children and youth in areas with socioeconomic challenges and in other areas for the use of LEL. However, this could change if the service is perceived as something exclusively for those who cannot afford to buy new equipment. In such a situation, the LEL brand might be associated with charity rather than with high-quality service catering to everyone, regardless of socioeconomic conditions. Consequently, a key factor is likely to ensure that the LEL brand continues to be perceived as representing high-quality and attractive services [24].

Our results also indicate that LEL reaches children and youth with a Low PAP, meaning that the group that is farthest from sports and PA. Visiting LEL does not seem to depend on either sports competence or experience, suggesting that those who choose to borrow equipment perceive the operation as more play- and recreation-oriented. The fact that the brand name includes ‘leisure’ rather than ‘sports’ may contribute to this perception. Additionally, the significant inclusion of equipment for activities such as play and outdoor recreation in the inventory may also contribute to this perception. From a public health perspective, this result should not be underestimated, as previous research has shown that interventions rarely succeed in reaching this specific target group [2, 14, 29], and the health benefits of increased PA are most significant among those who are least active [1–3].

The usage of borrowed equipment also suggests that the service is utilized primarily for play- and recreation-oriented activities rather than organized sports activities. Most respondents indicated that they use the equipment with people they already know, such as friends and family, engaging in play-oriented activities. This is a crucial piece of the puzzle in understanding which children and youth use LEL. If identification with the brand itself is the first step in using the service, the usage area can be seen as the second step. It is important for users to feel that they are allowed to use the equipment for the type of activities they desire. It is also noteworthy that only a small percentage use LEL for organized sports. This may be explained partly by the limited loan period (14 days) and partly by the fact that children and youth with a strong interest in sports do not identify with LEL services. However, this is something that should be further explored in future research.

Furthermore, according to our results, it is evident that LEL is significantly important to the group utilizing its services. Almost all respondents mentioned this themselves, and approximately half of them stated that they would have neglected the activity if they did not have the opportunity to borrow equipment. LEL also appears to be particularly crucial for older youth and those with low socioeconomic status. Socioeconomic factors likely play a role here, especially in terms of the possibility of alternative solutions, such as buying equipment instead. It is also conceivable that there is a particularly strong identification with LEL’s brand among individuals with low socioeconomic status. Aaker et al. argue that NPOs are perceived as more appealing brands by this group [24]. LEL also has clear marketing that signals democratic values of inclusion and equal opportunities to participate in sports and recreational activities. This may lead individuals with low socioeconomic status to perceive LEL services as accessible in a symbolic sense. The fact that most LELs are located in areas with low socioeconomic challenges likely contributes to individuals in these areas identifying with the brand [18, 21].

The provision of free lending services for sports and recreational equipment as a societal institution is a novel service that, to our knowledge, does not exist to a similar extent anywhere in the world. However, there are examples indicating a potentially emerging trend. In Norway, a service called ‘Bua’, which is quite similar to the LEL, is growing in scope each year. In Canada, as well as in Finland, traditional libraries have also begun to lend sports and recreational equipment to promote PA among the population. Therefore, it appears that sports and recreational equipment will soon become part of the sharing economy in many places around the world. Whether it evolves as part of public welfare services, as in Sweden and Norway, or as part of the commercial sharing economy, such as the ‘Library of Things’ in the UK, likely depends on the political and economic traditions within which the initiative develops. It is therefore important that researchers investigate how the operations need to be adapted to the specific contextual factors that affect implementation in each respective country. This includes how the brand of the service is perceived by potential users. Sports, leisure, and consumption culture in the context will affect which users identify with the service. The socioeconomic relationships between groups within the current societal context significantly influence the perception of the service. In a society characterized by greater inequality, such a service is likely to evolve into a form of charity, where materials are donated and subsequently utilized by individuals with limited economic resources. Regardless, this kind of service may potentially contribute to overcoming certain financial barriers [10, 14] that exist for engaging in PA.

The results from this study can contribute to the field interested in how innovative policy initiatives at the population level, initiated by public or non-profit actors, can enable increased PA for children and young people. In particular, the results are interesting for understanding how efforts to reduce economic barriers [12–14] can be received by the target group, based on the perception of the service offered [21]. Adding a sustainability perspective, which is increasingly important from a global climate standpoint, the results provide an indication of how a circular service can actually also contribute to increased PA.

### Limitations of the study

This study is limited to the operation of LELs during the summer period, which is important to consider when interpreting the results. During winter, other types of equipment are lent out, and it is highly likely that the composition of the group using LEL differs from that in summertime. It is therefore desirable to conduct a supplementary study for the winter season to provide a more comprehensive picture of the user base. Another limitation of the study is that we have not been able to fully rely on validated instruments. Since it is a partially unexplored area, such instruments have not been readily available. Moreover, the sample was deemed representative in many cases, except for a slight overrepresentation of youth aged 13–18 years and an underrepresentation of the oldest age group. The cross-sectional data in this study do not allow for statements regarding the causation of the importance of LELs for children’s and youths’ physical leisure activities. Future studies could benefit from employing a longitudinal design to enhance the understanding of how the use of LELs influences leisure-time activity levels over time.

### Implications for practice

The study’s results indicate that LELs currently reach a broad group of children and youth, suggesting that the service is perceived as accessible even to those who are not primarily interested in sports. However, the LEL brand is relatively unknown at present, and it remains an open question how perceptions among children and youth will evolve in the future. This is connected to the issue of whether LEL can build a strong relationship with its users [18, 21]. According to the concept of consumer-brand identification, individuals, when successfully identified, want to be associated with the brand they use [18]. Currently, the equipment provided by LEL is not marked to indicate its origin. It is conceivable that some individuals, such as those wanting to demonstrate environmental and climate awareness, wish to show that they have chosen not to consume new equipment, for example, when skiing or mountain biking, thereby demonstrating to others that it is possible to have an active lifestyle in a sustainable way. This may also apply to parents of younger children, who are likely to initiate visits to an LEL. However, it is also possible that the LEL does not succeed in building a strong brand, and it may not be desirable among users to showcase that the equipment is borrowed from an LEL [22]. For children and younger adolescents, the use of an LEL is likely influenced more by status, raising the question of how the LEL brand is perceived among peers and significant others [18, 23]. To promote positive identification, emotional bonds can be created with users, for example, through activities such as holiday- and break-time activities in which LEL engages and which create positive memories among users.

From a branding perspective, LEL also has the opportunity to position itself as a more recreation-oriented entity compared to organized sports [21, 24]. Our data suggest that LEL is currently used primarily for playful and recreational activities rather than formal sports. There is potentially a gap to fill, as the sports movement has faced criticism in prioritizing its own sporting interests over health work. Such a position could be advantageous for obtaining public support and marketing to potential users.

For a more equitable recreation sector, LEL should market itself in a way that resonates with children and youths from diverse socioeconomic backgrounds. This can be achieved by strategically placing stores in a variety of areas, including appealing urban centers. It is also crucial to ensure that the equipment is of high quality to establish the brand’s credibility among children and youths from more affluent backgrounds.

### Implications for policy

Local and national policymakers are advised to include financial support for LEL as part of public health initiatives, particularly given its potential to reach groups that are otherwise difficult to engage. The results support that the service reaches children and young people from different socioeconomic backgrounds as well as varying experiences with physical activity. Policymakers at various levels can initiate collaborations between LEL and different public services. For example, LEL can help increase access to recreational equipment in schools. Additionally, the health sector can collaborate with LEL to provide better access to materials and equipment, such as through physical activity referral schemes.

### Electronic supplementary material

Below is the link to the electronic supplementary material.

Supplementary Material 1

## Data Availability

The dataset generated and analyzed during the current study is not publicly available due to agreement with the Swedish Research Council for Sports Science, which commissioned the research group but is available from the corresponding author upon reasonable request.

## Abbreviations

LEL: Leisure Equipment Library [Swedish:‘Fritidsbanken’
PA: Physical activity
NPO: Nonprofit organization
PAP: Physical activity profile
