# Supplementary material for: Implementing a free lending of sports and leisure equipment service: a cross-sectional survey exploring user characteristics, utilization patterns, and significance among children and youth
Source: BMC Public Health. 2024 Jul 9;24:1826. doi: 10.1186/s12889-024-19339-3 (PMC11232254; doi:10.1186/s12889-024-19339-3)
Supplement: Supplementary file 1 — Supplementary Material 1 [file 12889_2024_19339_MOESM1_ESM.pdf]

1. Where do you answer this questionnaire?

☐ Inside a fritidsbank store

☐ Outside ("Fritidsbanken Pep-Up" or similar)

2. What fritidsbank are you at? (Write the name of city/town you are in)

4. What have you borrowed or returned today?

5. What are you going to use your borrowed material for? (You can choose several options if you want)

☐ Practice with my team/club

☐ Play with friends or family

☐ Sports activity with my friends (not in club)

☐ Practice or play by myself

☐ Play sports or play with people I do not know since before

☐ Other, write on the row below

6. Where are you going to use the material?

☐ In this area

☐ In another part of this city/town

☐ In another city/town

7. What would you have done if you had not been able to borrow equipment from Fritidsbanken?

☐ Ignored the activity

☐ Bought the material

☐ Borrowed the material from someone else

☐ Annat, skriv på raden

8. How important is Fritidsbanken for you to be able to do activities in your spare time?

☐ Not important at all

☐ Quite important

☐ Important

☐ Very important

Now follows two queations about what you do in your spare time

14. Do you play sports in club? (e.g. football in a team, athletics in an association, riding school)

☐ Yes

☐ No

15. Would you like to play sports in a club?

☐ Yes

☐ No

16. What do you do in your spare time? (You choose several options if you want)

☐ Go to the gym (e.g. group workout, weight training)

☐ Member of an outdoor association (e.g. The Scouts, Friluftsfrämjandet)

☐ Member of a gaming association (e.g. chess, board games, computer games)

☐ Dancing (e.g. in a dancing studio or association)

☐ Goes to theater school, cultural school, circus, play in a band

☐ Going to activities in a religious or cultural association

☐ I do nothing particular in my spare time

☐ Other, write on the row below

17. How often do you move so that you become short of breath or sweat? (e.g. sports, dancing, gym or similar)

☐ Never

☐ 1 time a month

☐ 1 time a week

☐ 2-3 times a week

☐ 4-6 times a week

☐ Every day

Now follows two statements about how you view yourself in relation to sports

18. Do you think you are good at sports?

- ☐ No, not good at all
- ☐ No, not so good
- ☐ Yes, pretty good
- ☐ Yes, very good

19. Compared to others your age, are you better or worse at sports?

- ☐ I am much worse
- ☐ I am little worse
- ☐ I am little better
- ☐ I am much better

Finally, some questions about you and your family situation.

20. Are you a...?

- ☐ Girl
- ☐ Boy
- ☐ Other, write in the row below

  ____

- ☐ Do not want to answer

21. How old are you?

____

22. What is the name of the street you live on? (Write the street you live on most of the time. Do not write the number of your house).

____

23. My family has plenty of money

- ☐ Strongly disagree
- ☐ Disagree
- ☐ Agree
- ☐ Strongly agree

24. What does your mother do for a living? (Write on the row below)

☐ My mother works with...

☐ I do not know
☐ My mother has no job right now
☐ My mother is studying
☐ I do not have a mother

25. What does your father do for a living? (Write on the row below)

☐ My father works with...

☐ I do not know
☐ My father has no job right now
☐ My father is studying
☐ I have no father

Is there anything you would like to add about Fritidsbanken? For instance, suggestion for improvements?

Do you want to receive a gift card for ice cream (value SEK 25) as a thank you for answering the questionnaire? Fill in your phone number below and you will receive a SMS with a digital gift card on your mobile phone. We will not use your phone number to contact you and we will remove all phone numbers after this study. The gift cards are sent out every weekday between 14.00-15.00.

Is it okay for us to contact you for a follow-up survey in approximately 6 months? (If you answer no, we will delete your phone number when you have received your gift card)

☐ Yes
☐ No
